# Sequestration of Vascular Endothelial Growth Factor (VEGF) Induces Late Restrictive Lung Disease

**DOI:** 10.1371/journal.pone.0148323

**Published:** 2016-02-10

**Authors:** Minna M. Wieck, Ryan G. Spurrier, Daniel E. Levin, Salvador Garcia Mojica, Michael J. Hiatt, Raghava Reddy, Xiaogang Hou, Sonia Navarro, Jooeun Lee, Amber Lundin, Barbara Driscoll, Tracy C. Grikscheit

**Affiliations:** 1 Division of Pediatric Surgery, Saban Research Institute, Children’s Hospital Los Angeles, Los Angeles, California, United States of America; 2 Developmental Biology and Regenerative Medicine Program, Saban Research Institute, Children’s Hospital Los Angeles, Los Angeles, California, United States of America; 3 Herman Ostrow School of Dentistry, University of Southern California, Los Angeles, California, United States of America; 4 Integrative Biology of Disease Graduate Program, Keck School of Medicine, University of Southern California, Los Angeles, California, United States of America; Medical College of Wisconsin, UNITED STATES

## Abstract

**Rationale:**

Neonatal respiratory distress syndrome is a restrictive lung disease characterized by surfactant deficiency. Decreased vascular endothelial growth factor (VEGF), which demonstrates important roles in angiogenesis and vasculogenesis, has been implicated in the pathogenesis of restrictive lung diseases. Current animal models investigating VEGF in the etiology and outcomes of RDS require premature delivery, hypoxia, anatomically or temporally limited inhibition, or other supplemental interventions. Consequently, little is known about the isolated effects of chronic VEGF inhibition, started at birth, on subsequent developing lung structure and function.

**Objectives:**

To determine whether inducible, mesenchyme-specific VEGF inhibition in the neonatal mouse lung results in long-term modulation of AECII and whole lung function.

**Methods:**

Triple transgenic mice expressing the soluble VEGF receptor sFlt-1 specifically in the mesenchyme (Dermo-1/rtTA/sFlt-1) were generated and compared to littermate controls at 3 months to determine the impact of neonatal downregulation of mesenchymal VEGF expression on lung structure, cell composition and function. Reduced tissue VEGF bioavailability has previously been demonstrated with this model.

**Measurements and Main Results:**

Triple transgenic mice demonstrated restrictive lung pathology. No differences in gross vascular development or protein levels of vascular endothelial markers was noted, but there was a significant decrease in perivascular smooth muscle and type I collagen. Mutants had decreased expression levels of surfactant protein C and hypoxia inducible factor 1-alpha without a difference in number of type II pneumocytes.

**Conclusions:**

These data show that mesenchyme-specific inhibition of VEGF in neonatal mice results in late restrictive disease, making this transgenic mouse a novel model for future investigations on the consequences of neonatal RDS and potential interventions.

## Introduction

Neonatal respiratory distress syndrome (RDS) is a restrictive lung disease of premature infants characterized by surfactant deficiency and structural lung immaturity that is often treated with supplemental pulmonary surfactant and mechanical ventilation. Multiple studies have implicated abnormal vascular endothelial growth factor (VEGF) signaling in the pathogenesis of several lung diseases including RDS in children, and chronic bronchitis and emphysema in adults [1–6]. Ventilation with periods of hyperoxia has been linked to reduced VEGF [7–10] and exogenous VEGF administration increases surfactant production and improves lung function [2,11]. Clinically, the long-term pulmonary consequences for preterm infants with respiratory difficulties are not well understood [12,13]. Because RDS occurs in conjunction with numerous confounders including premature birth, mechanical ventilation, multiple medications, and additional comorbidities, little is known about the isolated effects of postnatal pulmonary vascular disruption on subsequent lung development and function in humans. Animal models of RDS require similar interventions, which often preclude investigations into the late consequences of decreased VEGF. Therefore, we sought to study this in a novel mouse model of neonatal, inducible, mesenchyme-specific VEGF sequestration.

VEGF-A is an endothelial cell signal protein and a key mediator of angiogenesis and vasculogenesis [14], and mesodermal proliferation and differentiation into parabronchial smooth muscle, myofibroblasts, and other specialized cell types [15]. VEGF-A binds three receptors: VEGFR-1/Flt-1 (fins-liketyrosinekinase-1), VEGFR-2/Flk-1 (fetaliverkinase-1), and VEGFR-3/Flt-4. In full term infants without primary lung disease, immunohistochemical staining demonstrates persistent VEGF expression in bronchial epithelium and alveolar macrophages while its receptor Flt-1 appears in vascular endothelium and bronchial epithelium [3]. In mice, VEGF is expressed in lung mesenchyme and epithelium; its receptors are expressed on vascular and lymphatic endothelium [16]. This localization suggests that epithelium-mesenchyme crosstalk is crucial for normal differentiation of endothelial cells and neovascularization of tissues in a paracrine and cooperative manner [15,17].

Transgenic mice lacking functional VEGF, Flt-1 or Flk-1 demonstrate impaired vasculogenesis, derangement of endothelial cell differentiation/assembly and embryonic lethality [18–21]. This has necessitated the creation of alternative mouse models, short-term administration of antibodies and inhibitors, or deletion of regulators to investigate the function of VEGF in embryonic and newborn lung development [2,22–26] or adult respiratory disease pathogenesis [27–29]. However, the precise role of mesenchymal VEGF on postnatal lung development and the effect of chronic VEGF reduction on subsequent adult lung function are undefined.

The soluble form of Flt-1 (sFlt-1) is a high affinity, endogenous “decoy receptor” with low kinase activity that binds bioavailable VEGF, thereby reducing its activity without affecting underlying gene expression or Flk-1 binding [30]. We previously demonstrated that mesenchyme specific sFlt-1 expression reversibly attenuates whole body and organ specific growth in triple transgenic mice (dermo-1^Cre^- tetracycline reverse transcriptional activator (rtTA)^flox/flox^-tet(0)-sFlt-1) [31]. To determine whether chronic, mesenchyme-specific VEGF inhibition results in long-term modulation of alveolar epithelial type II cells (AECII) and whole lung function, we induced mesenchymal sFlt-1 expression and therefore, VEGF sequestration, from birth for 12 weeks. We hypothesized that mesenchyme-specific VEGF inhibition alone without barotrauma or other comorbid conditions would cause diffuse morphologic changes, resulting in persistent restrictive lung physiology.

## Methods

Additional detail for methods is provided in an online methods supplement (S1 File).

### Animals

All experiments were approved by the CHLA Institutional Animal Care and Use Committee. Triple transgenic mice expressing the soluble VEGF receptor, sFlt-1 in the mesenchyme (Dermo-1^Cre^-rtTA^flox/flox^-tet(0)-sFlt-1) were generated as previously published [31]. Littermate controls possessed neither the inducible sFlt-1 gene or the promoter. Dams were fed doxycycline chow at time of birth for transgene activation via breast milk. Once weaned, all mice were fed doxycycline chow until euthanasia at 3 months.

### Pulmonary function tests

Pulmonary function testing was performed on anesthetized mice using plethysmography via tracheostomy and forced pulmonary maneuvers (SCIREQ flexiVent, Montreal, Canada) as described in the online supplement. Lung volumes were corrected for total bodyweight.

### Tissue Morphology

Lungs were fixed under constant pressure of 25 cmH_2_0 and explanted as described in the online supplement. Lungs were fixed en bloc in 10% buffered formalin. The left lower lobe was embedded in paraffin. Samples were sectioned at 5μm and stained with hematoxylin and eosin.

### Mean Linear Intercept

For each lung, representative 20x magnification images without large airways or blood vessels were taken from the periphery of four different sections. A grid was superimposed and intercepts along both horizontal and vertical lines were counted to determine the average length of the acinar air space complex [32]. 20 images were counted per specimen.

### Detection of Apoptosis

Tissue sections were prepared according to manufacturer instructions by the In Situ Cell Death Detection (Fluorescein) kit (Roche Diagnostics). The total number of terminal deoxynucleotidyl transferase-mediated dUTP nick end labeling (TUNEL)-positive cells per total cross sectional area was counted by a blinded observer.

### Collagen stain

Slides were deparaffinized and stained using Sirius Red/Fast Green. Perivascular collagen was compared in tissue sections that were matched for lung location and vein lumen cross-sectional area. Five sections per animal were selected in a blinded fashion by a trained observer to minimize error associated with section selection. Stained collagen fiber was quantified using Fiji ImageJ [33] as described in the online supplement. Measurements of all sections from a single animal were averaged, and these averages were compared with unpaired, two-tailed student’s t-test.

### Hydroxyproline Assay

Hydroxyproline was quantified using a Colorimetric Assay Kit (BioVision, Milpitas, CA) per manufacturer-provided protocol on approximately 10mg of thawed lung tissue.

### Immunofluorescence

Sample slides were deparaffinized and hydrated. Antigen retrieval was performed with 10mM Na-Citrate (pH 6.0). After blocking in TBS/0.2% Tween^®^20/30% Bovine Serum Albumin, slides were incubated with primary antibodies and fluorescently labeled secondary antibodies as specified in the online supplement. After mounting with Vectashield^®^ medium containing 4',6-diamidino-2-phenylindole (DAPI), a blinded, trained observer or public domain ImageJ software (nih.gov) counted cells per 20X microscopic field.

### Western blot analysis

Freshly prepared lung tissue was stored at -80°C prior to isolation of protein from the right lower lobe. After Western blotting, primary antibodies (listed in the online supplement) were applied to membranes, followed by washing and application of horseradish-peroxidase-labeled secondary antibodies. Chemiluminescent signals were detected using SuperSignal WestPico reagent (Thermo Scientific), scanned and normalized to ß-actin expression, then analyzed using ImageJ.

### Polymerase Chain Reaction

RNA was extracted from 2–5 mg fresh lung tissue and purified with the RNeasy Mini Kit (Qiagen Inc, Valencia, CA). Reverse transcription was performed by incubating 200 ng of RNA with iScript^™^ Reverse Transcription Supermix (Bio-Rad, Hercules, CA). qPCR was performed with SYBR^®^ Green Master Mix (Roche). Relative expression of target genes was then compared between mutants and littermates.

### Statistics

All p values were obtained using unpaired, two-tailed Student’s T-Tests unless otherwise indicated (Excel, Microsoft Corporation, Redmond, WA, USA). Data are represented as the mean ± standard deviation (SD) unless otherwise stated.

A p-value of <0.05 was considered significant.

## Results

Twelve Dermo-1^Cre^-rtTA^flox/flox^-tet (0)-sFlt-1 transgenic mutant mice and thirteen littermate (LM) controls were induced by doxycycline chow given to both mother and pups beginning at birth. At three months of age, mutant mice demonstrated lower body weights compared to LM controls (35.1±1.5 g v. 17.5±1.4 g, n = 8, p<0.001). A three fold increase in sFlt-1 expression and concomitant 10-fold decrease in tissue VEGF levels were previously confirmed through PCR and ELISA assay, respectively [31].

**Fig 1:**
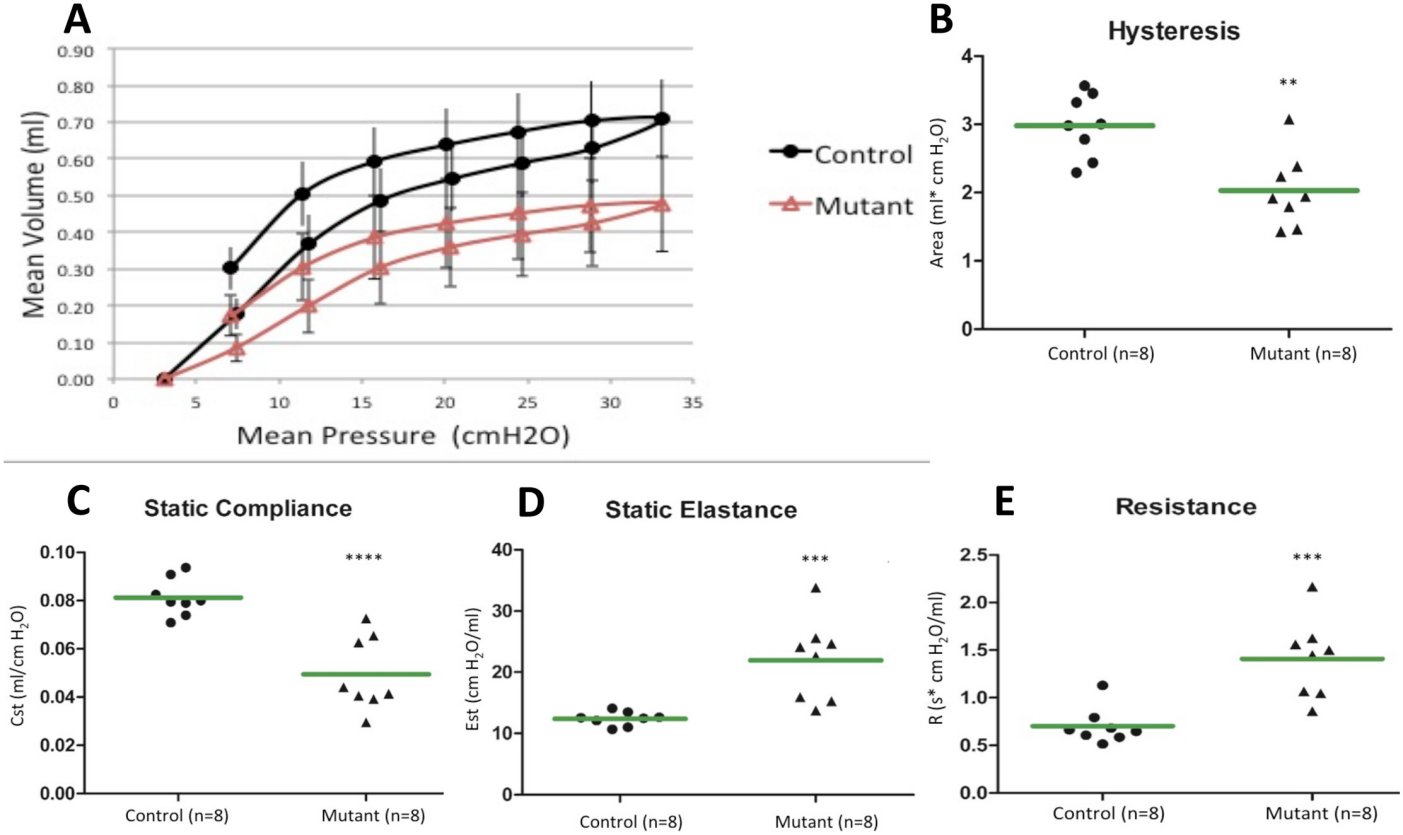
Pulmonary function tests of mutant mice with reduced bioavailable VEGF indicate a restrictive lung disease. Plethysmography was performed at 12 weeks on anesthetized mutant mice and littermate controls. (A) Pressure volume curves of mutant mice were shifted downward compared to control mice. Data are expressed as mean ± SEM. (B, C) Mutant mice also demonstrated a 30% decrease in hysteresis and compliance. (D, E) They also showed a 1.5-fold increase in static elastance and a 2-fold increase in resistance, all indicative of a restrictive lung disease. All differences were statistically significant. Green bars represent mean values, **P<0.01, ***P<0.001, ****P<0.0001 compared to controls.

Pulmonary function was evaluated by plethysmography at twelve weeks on terminally anesthetized animals. Lung volumes were corrected for body weight. The resulting pressure-volume curves of the mutant mice were shifted downward along the volume axis indicating a restrictive lung disease pattern (Fig 1A). As pressure was incrementally varied, the mutant lungs inflated to a lesser volume when compared to LM controls. In concordance with the depressed pressure-volume loops, mutant lungs demonstrated a statistically significant decrease in hysteresis in comparison to controls (Fig 1B, Table 1). Furthermore, mutant lungs showed decreased static compliance (Fig 1C, Table 1) as well as increases in static elastance (Fig 1D, Table 1) and dynamic resistance (Fig 1E, Table 1), as expected for restrictive lung disease. All other pulmonary function parameters evaluated were statistically different between controls and mutants, except tissue hysteresivity (Table 1).

**Table 1:** Pulmonary function parameters (n = 8).

Parameter (unit)	LM control	Mutant	P-value
Hysteresis (ml*cmH_2_0)	2.98±0.63	2.033±0.57	0.002
Static compliance (ml/cmH20)	0.081±0.0075	0.049±0.0147	0.0001
Static elastance (cmH20/ml)	12.4±1.127	21.99±6.451	0.0014
Dynamic resistance (cmH20*s/ml)	0.642±0.084	1.41±0.398	0.0006
Tissue hysteresivity (ml/cmH20)	0.215±0.0488	0.204±0.0154	0.575
Salazar Knowles curvature parameter of deflation (cmH20^-1^)	0.144±0.0060	0.118±0.01157	0.0001
Dynamic elastance (cmH20/ml)	25.26±2.455	59.14±18.43	0.0001
Dynamic compliance (ml/cmH20)	0.040±0.0040	0.019±0.0063	0.0001
Newtonian resistance of the central airways (cmH20*s/ml)	0.316±0.064	0.383±0.055	0.0476
Tissue damping (cmH20/ml)	5.032±1.026	12.16±4.12	0.0007
Tissue elastance (cmH20/ml)	23.74±2.54	59.29±18.95	0.0001

**Fig 2:**
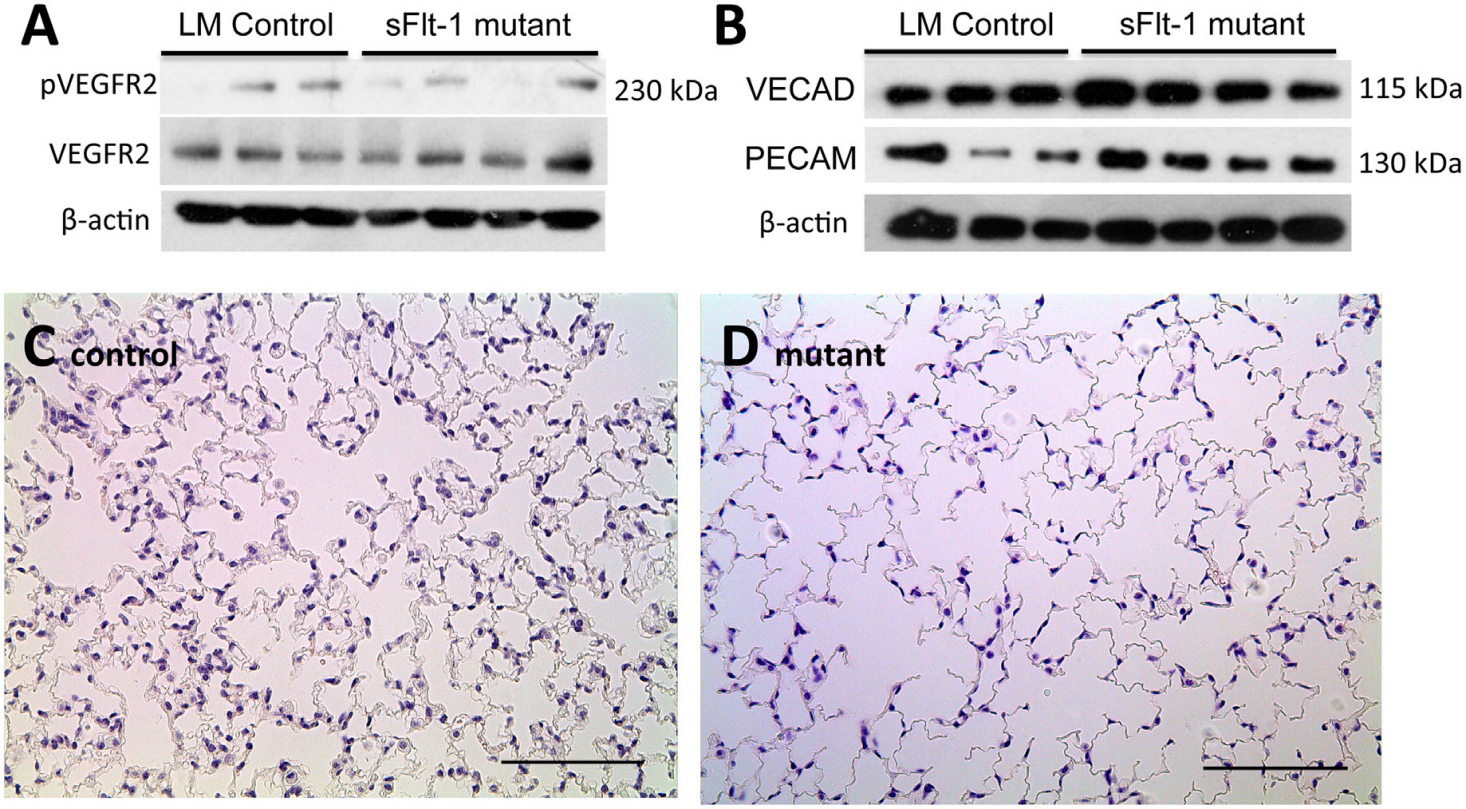
Mesenchymal VEGF sequestration does not affect pulmonary angiogenesis but affects acinar air space morphogenesis. Western blot analyses of (A) total VEGFR-2 and phosphorylated VEGFR-2, and (B) endothelial markers PECAM-1 and VE-cad. No significant difference in expression between control and mutant mice was noted on either analysis. (C-D) Representative H&E images of peripheral lung sections from control and mutant mice demonstrate larger acinar air spaces in mutants. This was confirmed by mean linear intercept analysis. Images at 20x magnification, scale bars represent 100 μm.

To determine the effect of mesenchymal VEGF sequestration on angiogenesis, we evaluated the activation of VEGFR-2, which is believed to play a greater role in endothelial cell differentiation and proliferation [34]. Western Blot analysis did not show a significant decrease in phosphorylated VEGFR-2 levels relative to total VEGFR-2 levels (0.74±0.53 v. 0.38± 0.21, n = 3, p = 0.26) (Fig 2A). Furthermore, PCR quantification of endothelial-specific markers platelet endothelial cell adhesion molecule (PECAM-1) revealed no significant difference in gene expression of PECAM-1 (1.93±0.324 v. 1.50±0.54, n = 3, p = 0.28) relative to glyceraldehyde-3-phosphate dehydrogenase (GAPDH) expression. Western blot analysis also revealed no significant difference in relative expression of PECAM-1 protein levels (0.53±0.25 v. 0.69±0.17, n = 3, p = 0.38) or vascular endothelial cadherin (VE-cad) (0.49±0.07 v. 0.75±0.18, n = 3, p = 0.08) corrected to β-actin (Fig 2B). Nor was there a significant difference in the cell turnover of endothelial cells as measured by the number of TUNEL+/PECAM+ cells per unit area (0.579±0.459 cells/mm^2^ vs 0.869±0.532 cells/mm^2^, n = 7 LM; 6 mutant, p = 0.67) or percentage of PECAM+ cells per total number of TUNEL+ cells (0.77±0.20% vs 0.62±0.20%, n = 7, p = 0.14).

VEGF also affects acinar air space morphogenesis and so these structures were also evaluated. At 12 weeks, tissue was obtained from anatomically matched segments of the lower left lobe. The alveolar spaces of both control and mutant lungs were comparable and appropriately inflated. Comparison of alveolar-ductal air space size by mean linear intercept analysis demonstrated significantly larger airspaces in mutant lungs (66.27±1.48μm vs 75.94±0.97μm, n = 3, p<0.001) (Fig 2C control and Fig 2D mutant).

**Fig 3:**
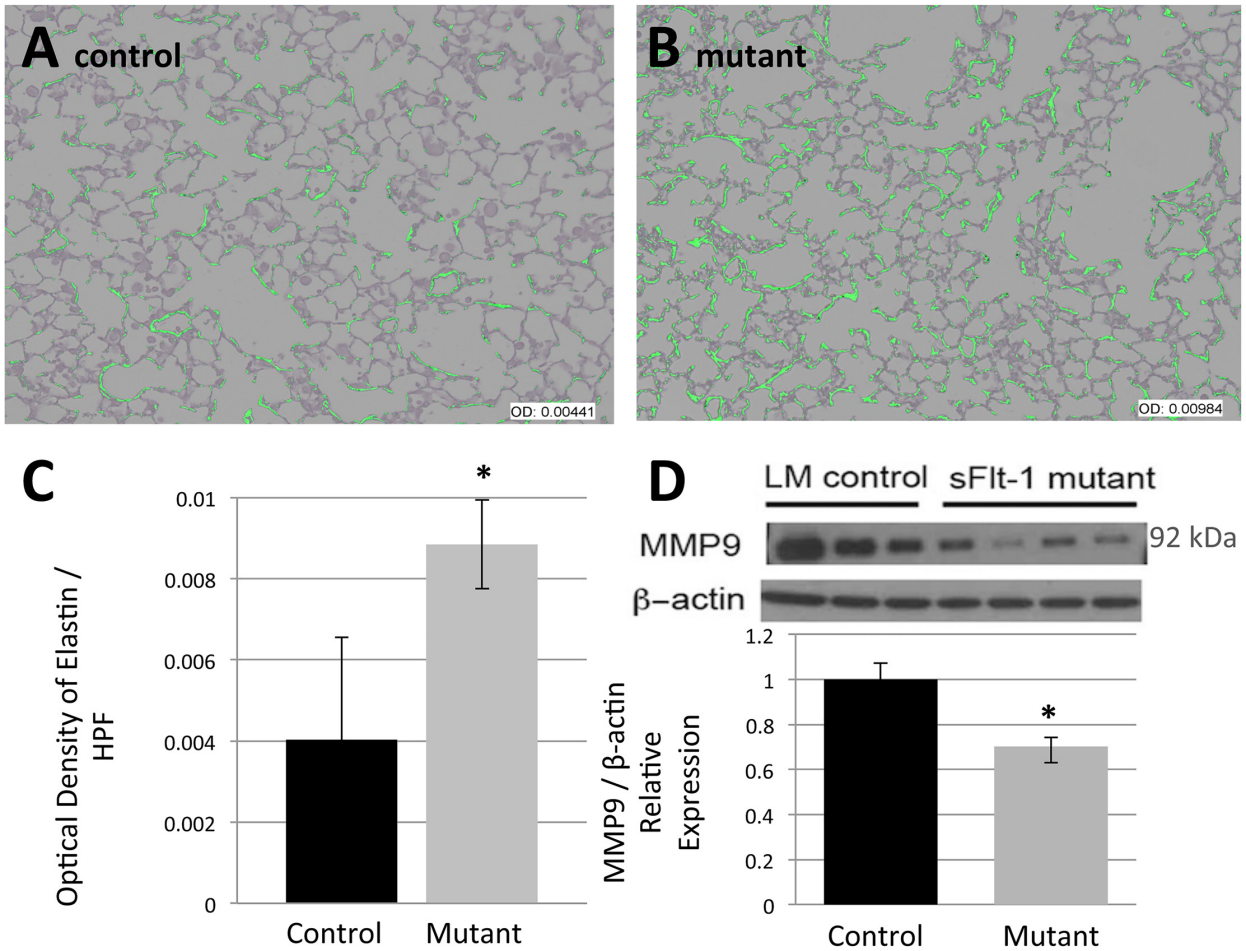
Reduced VEGF decreases MMP9 expression and consequently, increases pulmonary extracellular matrix elastin. Analysis was performed on anatomically matched distal left lobe segments that consisted predominantly of alveoli. (A-B) Representative images of an overlay mask of elastin staining show more prominent elastin in the alveolar septae of mutant mice compared to control mice. (C) Optical density quantification of elastin confirms this statistically significant increase in mutant mice. (D) Western blot analysis for matrix metalloproteinase 9 (MMP9) demonstrates a statistically significant decrease in MMP9 expression in mutant mice. Scale bars represent 50 μm. Data are expressed as mean ± SD, *P<0.05 versus control.

To assess the effects of VEGF sequestration on the composition of extracellular matrix protein in distal lung, Hart’s method was performed on matched samples to stain for elastin. All sections analyzed were predominantly composed of alveoli and were free of confounding structures such as conducting airways and vessels. Elastin fibers were more prominent along the alveolar septae and throughout the distal lung of mutants compared to controls. Computer assisted image processing was employed to create on overlay mask of positive elastin staining (Fig 3A control and Fig 3B mutant) which demonstrated the increased prominence of septal elastin in mutant mice. Quantification confirmed a significant increase in optical density (OD) of elastin stain per high power field among mutants (OD 0.004033±0.002514 v. 0.008849±0.001097, n = 5, p = 0.038) (Fig 3C). To assess whether the relative excess of elastin resulted from inhibited degradation, Western blot analysis for the prominent ECM degradation enzyme matrix metalloproteinase 9 (MMP9) was performed. Relative MMP9 protein expression was significantly lower in mutants (1±0.073 v. 0.702±0.041, n = 3 LM; 4 mutants, p = 0.012) (Fig 3D). This is consistent with a described induction of MMP9 expression by VEGF [35] that is lacking in experimental mutants.

**Fig 4:**
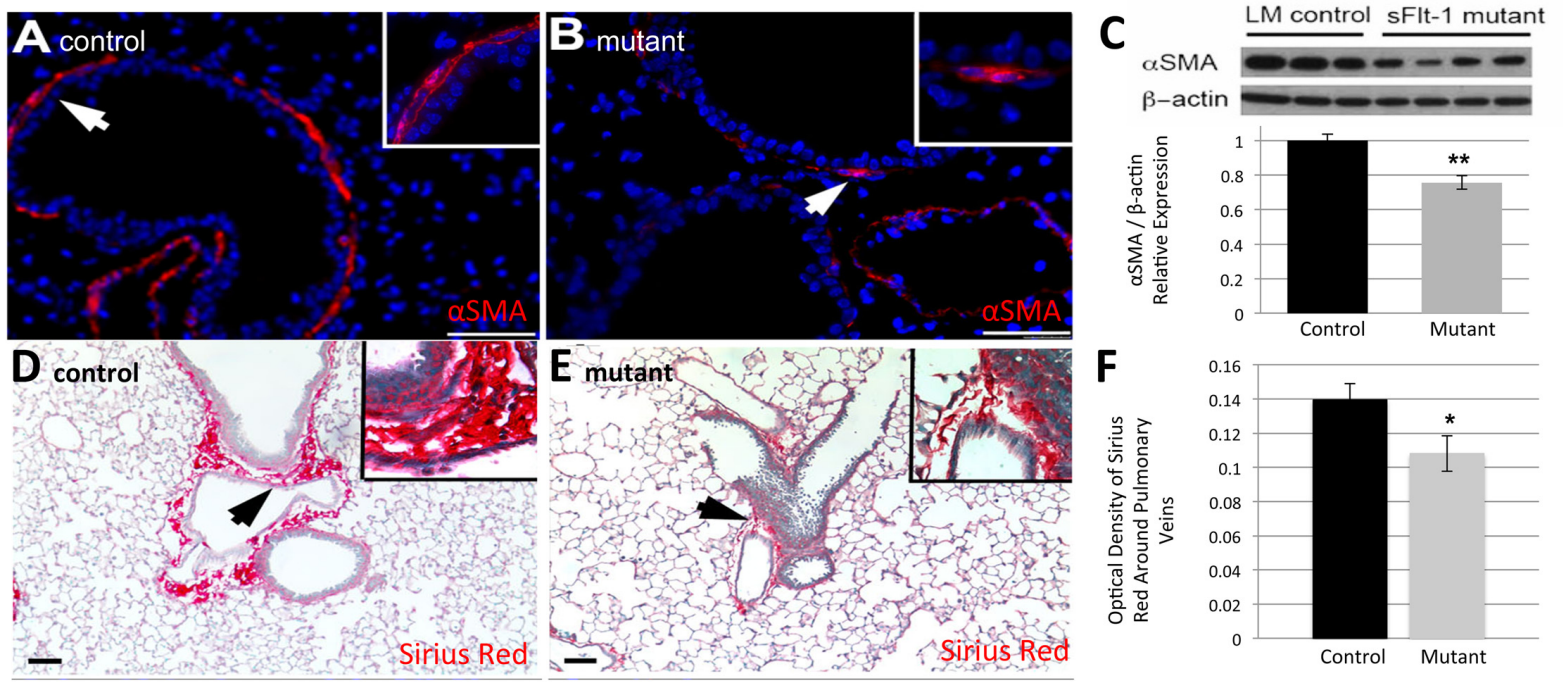
Reduced VEGF disrupts the parenchyma around airways and vasculature. (A, B) Representative images of immunofluorescent staining for α smooth muscle actin (αSMA) reveals fewer putative myofibroblasts surrounding the airways in mutant mice (B) compared to controls (A). (C) Quantification by Western blot confirms a statistically significant decrease in the relative abundance of αSMA in mutants. (D, E) Representative images of Sirius red stain for Type 1 collagen demonstrates decreased peri-vascular staining in mutants (E) compared to controls (D). (F) Mutant mice express significantly less collagen around pulmonary veins based on optical density quantification. *White and black arrows* point to magnified areas shown in insets. Scale bars represent 50 μm. Data are expressed as mean ± SD, *P< 0.05, **P< 0.01 versus control.

In addition to the differences in alveolar architecture, immunostaining demonstrated significant differences in airway support. Immunofluorescent staining for alpha smooth muscle actin (αSMA) revealed fewer putative myofibroblasts subjacent to the bronchioles in the terminal and segmental airways of mutants (Fig 4B) compared to controls (Fig 4A). Western blot analysis confirmed a decrease in the relative abundance of αSMA protein in mutants (1±0.037 v. 0.755±0.043, n = 3 LM; 4 mutants, p = 0.009) (Fig 4C). Sirius Red/Fast Green collagen staining showed a gross decrease in type 1 collagen in mutants (Fig 4E) compared to controls (Fig 4D), particularly surrounding the pulmonary veins near bronchioles. In addition, the collagen had a thin and disorganized appearance (Fig 4E inset). Quantification of Sirius red-stained collagen via OD was not significantly different in the airways or alveoli between the two groups as measured per high power field (OD 0.089±0.028 v. 0.080±0.037, n = 6, p = 0.26). However, the area immediately surrounding size-matched pulmonary veins of mutant mice showed a statistically significant decrease in collagen quantity compared to controls (OD 0.138±0.042 v. 0.108±0.010, n = 6, p = 0.030) (Fig 4F). There was no difference in hydroxyproline levels from whole lung (28±14 pg/mg v. 46±13 pg/mg, n = 3 LM; 4 mutants, p = 0.14), indicating that the observed difference in peri-venous collagen deposition was a localized phenomenon.

**Fig 5:**
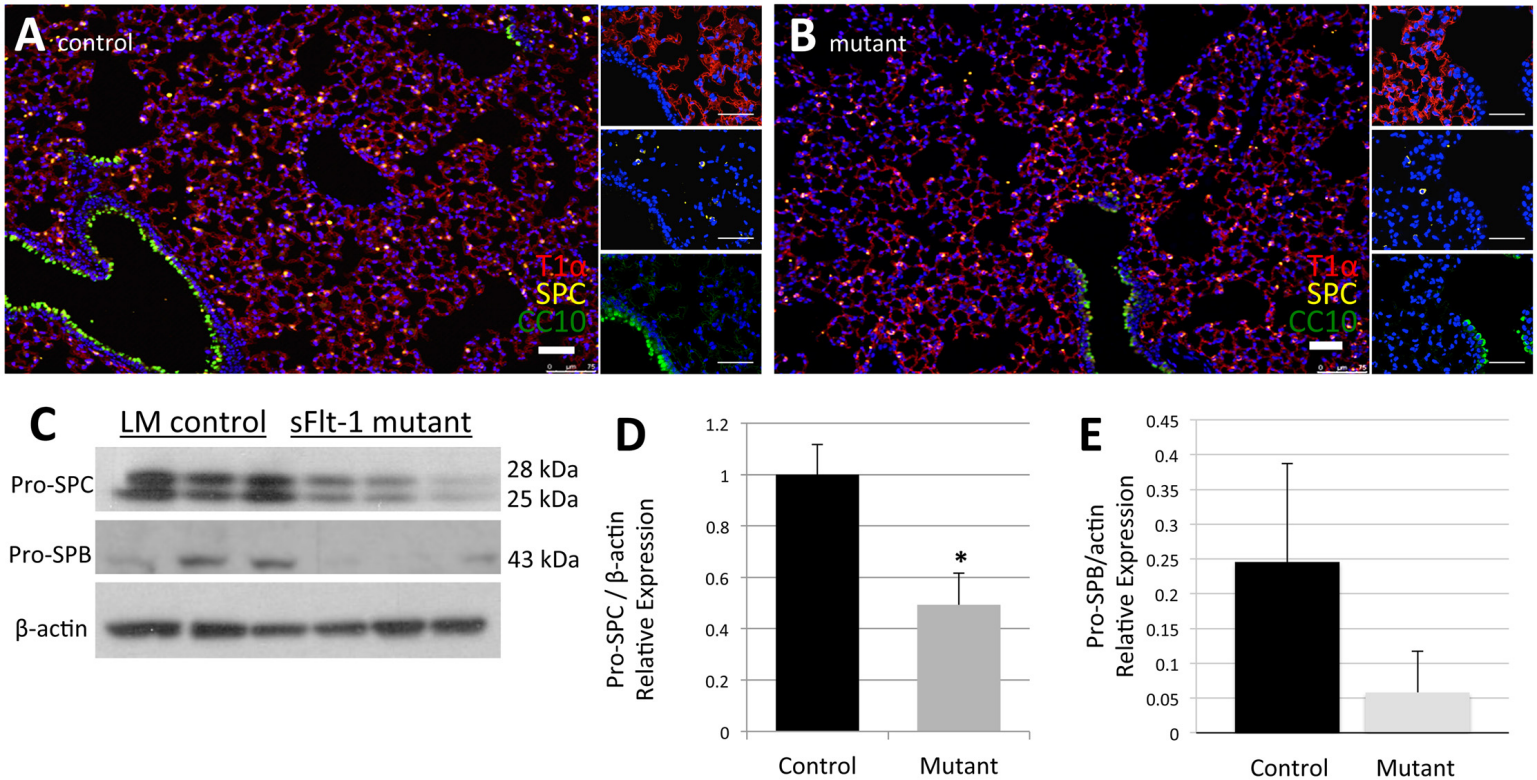
Reduced VEGF lowers surfactant production. (A, B) Representative images of immunofluorescence staining for cell specific markers T1α (Type I pneumocytes), SPC (Type II pneumocytes), and CC10 (Club cells) show similar distribution, frequency, and percentage of each cell type in both control mice (A) and mutant mice (B). (C) Western blot data for pro-SPC and pro-SPB. (D) Mutant mice produce significantly less pro-SPC, a downstream target of VEGF-R activation. (E) Decreased levels of pro-SPB seen in mutant mice were not significant. Scale bars represent 50 μm. Data are expressed as mean ± SD, *P<0.05.

Immunofluorescence staining for cell specific markers confirmed the presence of type I pneumocytes (AECI by T1α), type II pneumocytes (AECII by SPC), and Club cells (by CC10) in both groups (Fig 5A and 5B). Each cell type was observed in the expected distribution and frequency. By computer-assisted quantification of total cells per high power field and percentage of AECII, we observed a mean of 1,412±221 total cells per high power field among controls. This was not significantly different from the mutants, which averaged 1,465±298 cells per high power field (n = 13 LM; 12 mutants, p = 0.618). The percentage of SPC+ AECII, was also similar (11.53±5.71% v. 9.27±3.69%, n = 13 LM; 12 mutants, p = 0.257). However, even without a significant change in the percentage of AECII, we observed a significant decrease in relative pro-SPC expression by Western Blot in the mutants (1±0.12 v. 0.49±0.12, n = 3, p = 0.04). A decrease in relative pro-surfactant protein B (SPB) was also noted on Western Blot, but this difference was not significant (0.25±0.14 v. 0.06±0.06, n = 3, p = 0.10) (Fig 5C–5E).

**Fig 6:**
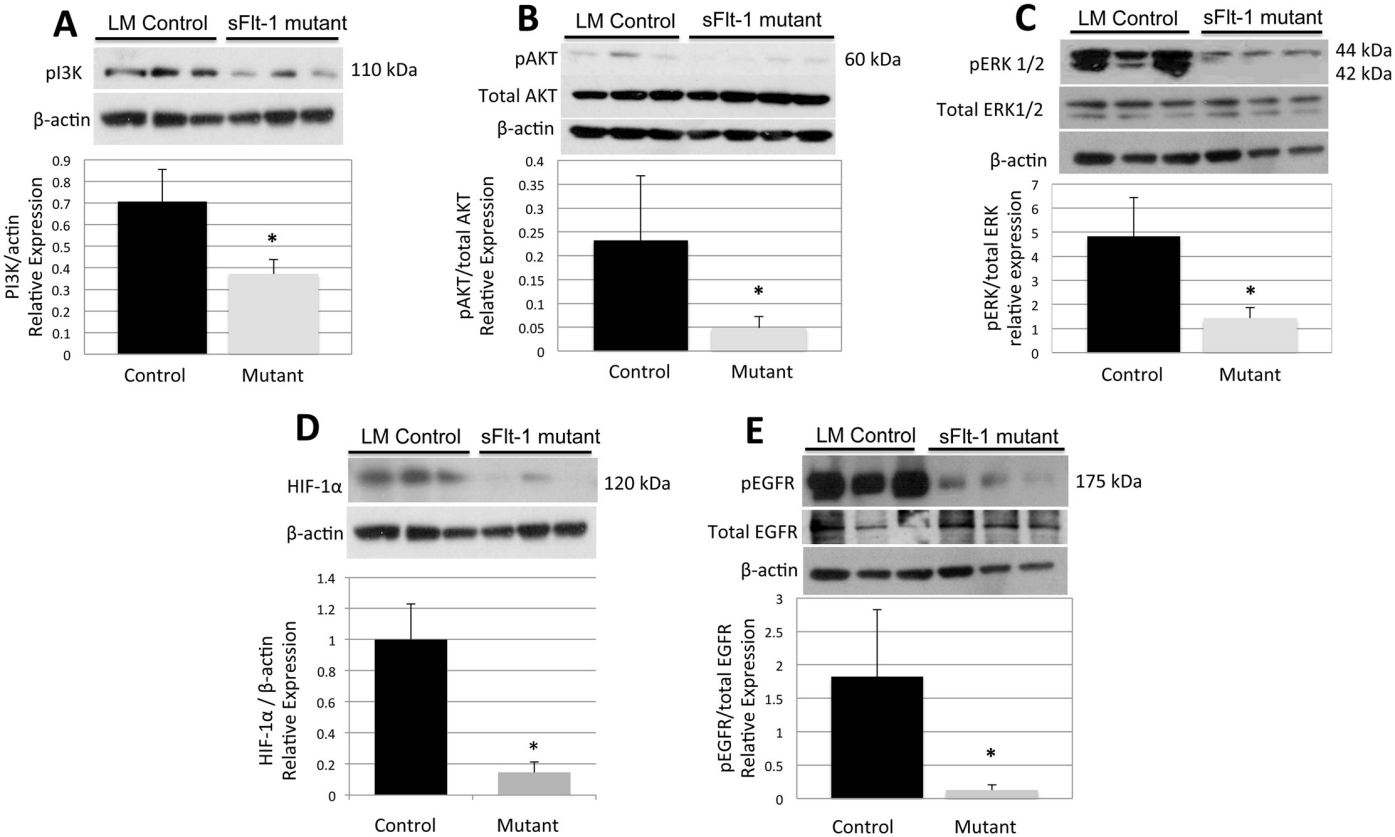
VEGF sequestration decreases PI3K-Akt and MAPK-ERK pathway signaling. Western blot data for (A) PI3K, (B) phosphorylated and total Akt, and (C) phosphorylated and total ERK1/2 demonstrate decreased activation of these signaling pathways in mutants. (D) Western blot data also show decreased relative production of HIF-1α in mutant mice. (E) Mutant mice also express decreased relative levels of activated EGFR, which could further decrease surfactant production through its synergistic effect on VEGF expression. Data are expressed as mean ± SD, *P<0.05 vs control.

This impaired ability to produce surfactant despite a normal number and distribution of AECII merited further evaluation. Therefore Western blot analysis was carried out on multiple candidate pathways with the goal of defining a potential mechanism for the observed pneumocyte dysfunction. Analysis showed a significant decrease in relative expression of PI3K (0.71±0.14 v. 0.37±0.06, n = 3, p = 0.02), phosphorylated Akt (0.23±0.14 vs 0.05±0.02), and phosphorylated ERK1/2 (4.82±1.6 v. 1.42±0.46, n = 3, p = 0.02) compared to total levels of each respective protein (Fig 6A–6C). Growth factor activation of both PI3K-Akt pathways and MAPK-ERK pathways can induce expression of hypoxia inducible factor 1, alpha subunit (HIF-1α) protein. Mutants with decreased PI3k-Akt and MAPK-ERK signaling also showed marked attenuation in the relative expression of HIF-1α (1±0.230 v. 0.146±0.068, n = 3, p = 0.023) (Fig 6D), which can lead to reduced surfactant production.

VEGF signaling via the MAPK-ERK pathway has a known downstream effect on SPC. Epidermal growth factor (EGF) signaling via epidermal growth factor receptor (EGFR) is another known pathway affecting surfactant production and VEGF expression through an ERK 1/2 dependent pathway, and EGFR was significantly less activated in mutant lungs, with a near fifteen-fold decrease in phosphorylation of EGFR (1.82±1.00 v. 0.13±0.08, n = 3, p = 0.04) relative to total EGFR levels (Fig 6E). Together, these factors suggest that although a normal number of AECII exist, VEGF and EGFR signaling via ERK may contribute to decreased surfactant production.

## Discussion

**Fig 7:**
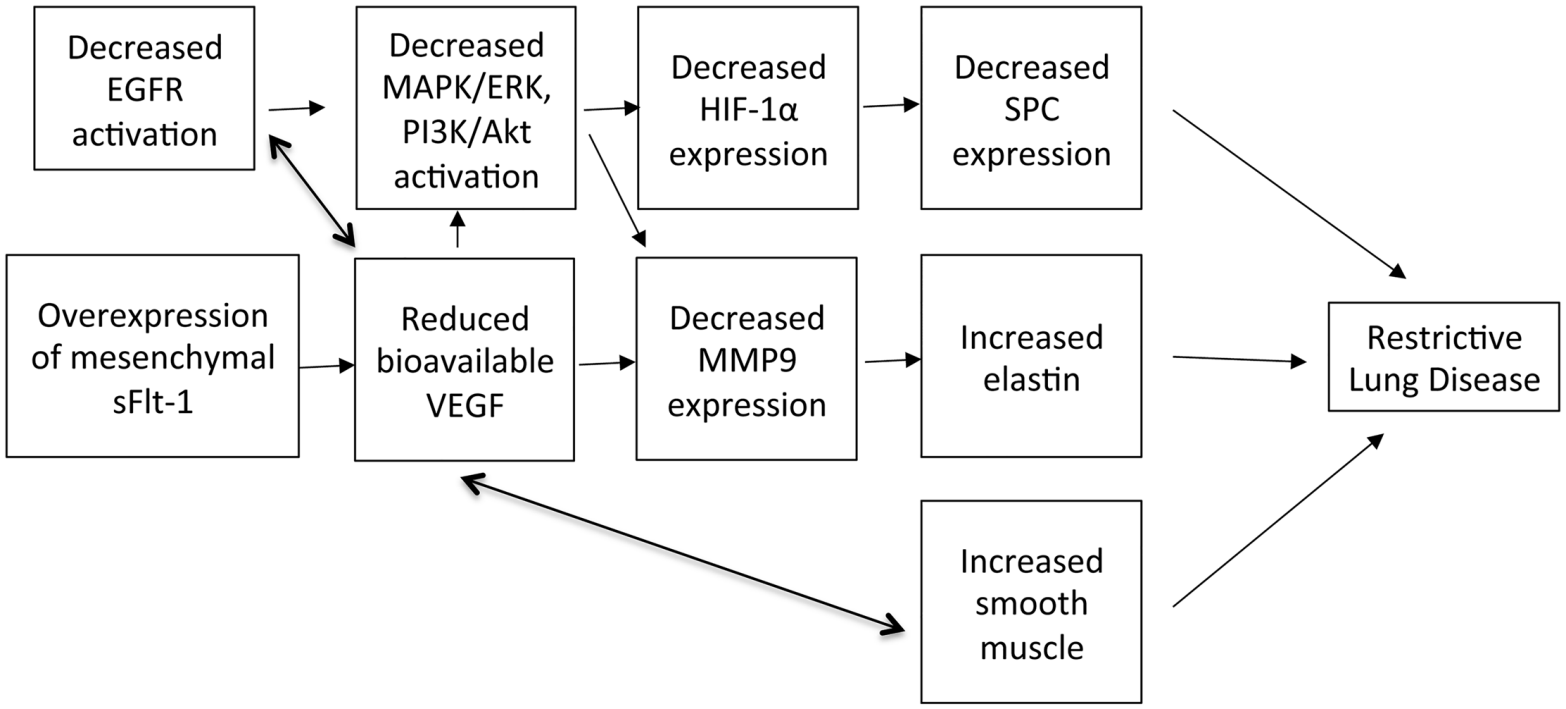
Summary of effects of VEGF sequestration leading to restrictive lung disease phenotype.

VEGF sequestration by the mesenchymal sFlt transgene causes a decrease in surfactant production, increased parenchymal elastin and smooth muscle, and a decrease in alveolarization that culminate in a late restrictive lung disease pattern (Fig 7) without grossly affecting angiogenesis. This is a novel transgenic mouse model for investigating the sustained and isolated effects of VEGF on neonatal lung development and maintenance of proper lung function through adulthood. Furthermore, this is a survivable phenotype with mutant mice demonstrating lower weights but appropriate alveolar epithelial cell differentiation, quantity, and distribution. As seen in a previous study, the systemic mesenchymal sFlt-1 sequestration of VEGF does generate smaller mutant mice, possibly due to systemic abnormalities in nutrient and growth factor delivery, and resultant organ growth [31].

Interestingly, despite the reduction in mesenchymal VEGF, there is no clear effect on VEGFR-2 signaling or subsequent vascular development in adult mutant lungs. Highest expression of VEGFR-2 occurs on vascular endothelial cells during embryonic vasculogenesis and angiogenesis, and during active angiogenesis in adults, e.g. in the uterus or neoplasms [34]. In adult lungs of mice, however, VEGFR-2 is constitutively activated and may act more as a pro-survival and maintenance factor [36]. Consequently, a difference in VEGF bioavailability would not be expected to have a significant effect on VEGFR-2 activation in our adult mutant mice.

Since VEGFR-2 is believed to play a larger role in transducing VEGF’s effects on endothelial cells, it follows that no differences in expression of vascular markers PECAM-1 and VE-cad were noted in our mutants. In mouse embryos, selective epithelial VEGF inactivation causes a dose dependent decrease in PECAM-1 and an almost complete absence of pulmonary capillaries [22,23]. Inducing the VEGF sequestration postnatally in our mutant mice may have bypassed the period when VEGF sequestration has the greatest effect on angiogenesis and vasculogenesis. Additionally, VEGF is only one of several important angiogenic factors and in the setting of chronic VEGF inhibition, elevated expression of other angiogenic factors may compensate for sequestration of mesenchymal VEGF. Alternatively, epithelial and mesenchymal VEGF may have complementary but different roles in pulmonary angiogenesis, development, and remodeling. We observed decreased perivascular collagen (Fig 4E–4G) and additional disruptions at the level of the capillary, such as barrier function, diffusion capacity, or other functional parameters, may also exist, undetected by our approach.

Although the vasculature remains grossly normal, reduced mesenchymal VEGF alters non-vascular components of the lung. Mutants appeared to have impaired alveolarization (Fig 2C and 2D), which has been described previously with VEGF blockade [37,38]. Mutants also demonstrated abnormal extracellular matrix with increased elastin (Fig 3A and 3B), decreased myofibroblasts (an intrinsic source of VEGF) (Fig 4A and 4B), and reduced levels of αSMA (Fig 4C and 4D). These effects suggest that mesenchymal sequestration in the postnatal period have more specific effects on alveolarization and parenchymal development than on vascular development. Consequently, the localization and timing of VEGF inhibition may affect various formative processes disparately. Manipulating the period of VEGF inhibition with this inducible and reversible model will enable further elucidation of VEGF’s effects at different developmental stages.

In our model, VEGF directly affects the extracellular matrix of the lung parenchyma via MMPs. The intimate relationship and feedback between VEGF and MMPs is well described [35,39,40] in many tissues including the lung. In studies of human tumor angiogenesis, VEGF and MMP9 levels have been shown to correlate, with each able to potentiate the other [39,40]. Our model is consistent with this described relationship, as both VEGF and MMP9 levels are lower in mutants (Fig 3D), likely representing a primary perturbation of that pathway. This relative absence of metalloproteinase, one of the primary degradation factors for elastin [41], may explain the observed increase in elastin deposition (Fig 3A and 3B). Such increased ECM protein deposition in mutants could certainly contribute to the observed restrictive lung disease phenotype (Fig 1). Many of these proteins are part of multiply crosstalking signaling webs and therefore perturbations like the decreased levels of MMP9 in the mutant lung tissue may also represent a feedback mechanism for preservation of the sparse remaining perivascular collagen, as previously described in both murine and human models [42,43], with the additional effect of attenuating elastin degradation.

In preterm infants with RDS, the restrictive respiratory physiology is the result of a deficiency of pulmonary surfactant and the subsequent reduction in compliance and increase in surface tension. However, this outcome always occurs within the context of multiple additional factors, including preterm delivery, mechanical ventilation/barotrauma, medications, other primary medical problems, and varied systemic oxygenation levels. Randomized control trials have demonstrated that surfactant therapy decreases the morbidity and mortality of RDS, making surfactant the mainstay of RDS treatment [44]. Qualitative or quantitative surfactant defects also contribute to other acute and chronic lung diseases associated with abnormal extracellular matrix and lung function, such as interstitial pulmonary fibrosis [45]. Importantly, our data show that mesenchymal VEGF sequestration alone results in decreased surfactant protein expression (Fig 5C–5E) and concomitant restrictive lung disease. This is consistent with known effects of VEGF on surfactant production in in vitro cell cultures and in vivo models with the addition of preterm delivery [2,11]. In this transgenic model, however, the phenotype is induced and maintained postnatally in full term pups and adult mice, without the addition of other interventions.

Analysis of various signaling pathways in the mutant mice points to a possible mechanism for the surfactant deficiency and consequently, offers other points of potential therapeutic intervention. Synthesis of surfactant proteins has been shown to be driven by a MEK1/2 –ERK1/2 pathway [46,47] and ERK downregulation attenuates expression of both surfactant proteins [15,48] and MMP9 [49]. The ERK pathway was downregulated in our mutant mice (Fig 1C) and correlates with the observed changes in pulmonary surfactant production. Both VEGF and EGF can activate this pathway and perturbation of this critical signaling nexus could be the mechanism that decreases surfactant protein expression and presumably surfactant production. We speculate that a primary driver for the disturbance of this pathway is decreased VEGF because of the known synergy between VEGFR and EGFR [50,51].

Despite the changes in body size and pulmonary function induced by VEGF sequestration, any resultant hypoxia was not significant enough to drive HIF-1α expression (Fig 6D). Under normoxic conditions, activation of receptors with tyrosine kinase activity, including EGFR, has been reported to induce HIF-1α expression through the PI3K/AKT pathway [51–53]. Consequently, decreased PI3K/Akt activation may have been the primary abnormality driving reduced HIF-1α production. Alternatively, the reduced levels of HIF-1α may have been due to the failure of VEGF sequestration to induce severe enough hypoxia, compounded by the nearly absent upstream EGFR signaling (Fig 4D). As HIF-1α is one of the primary drivers of VEGF expression and surfactant production [54], this reduction in HIF-1α further exacerbates the decrease in VEGF and surfactant proteins seen in mutant mice.

Notably, diffuse interstitial fibrosis and enlarged air spaces have been noted in some pediatric patients with mutations in the gene encoding SP-C, *SFTPC* [55] [56]. Whereas mutations in SP-B often present as severe or fatal neonatal lung disease, mutations in SP-C typically present later as chronic interstitial lung disease [57]. Although specific histologic findings may vary, affected lungs contain abnormal alveolar walls affected by fibrosis and inflammation which lead to restrictive lung function as seen in our model [58]. Although SP-C deficient mice also demonstrate enlarged airspaces and interstitial thickening, their pulmonary function tests are more consistent with emphysema rather than the restrictive phenotype of our mice [59]. They also have increased MMP-9 expression with increased smooth muscle expression in comparison to the decreased levels we see in our mice. These differences highlight that effects of VEGF inhibition are not due to decreased SP-C expression alone.

This study has limitations. Analyzing whole lung tissue does not allow for the differentiation of which cell types contribute to the observed protein expression changes. However, in this model, though a moderate reduction peribronchial myofibroblasts is observed, there are no observed changes in overall epithelial cell numbers or ratios of individual epithelial cell types. Other potential confounders include unknown contributions by immune cells, particularly macrophages, which are known to be capable of altering the ECM with their own proteinases.

Clinically, these data support previous studies that suggested reduced VEGF levels could be a useful marker for infants at risk for RDS and that exogenous VEGF may help improve surfactant production. Other activators of the MEK1/2-ERK1/2 pathway, such as EGF or mechanical stretch may be potential therapies as well [47]. Additionally, given the deleterious effects of reduced VEGF on surfactant production, these data underlie the importance of careful ventilator management, as hyperoxia suppresses VEGF expression.

Long-term, mesenchyme-specific VEGF inhibition induces surfactant-deficient, restrictive lung disease in the developing mouse lung, independent of prematurity, oxygenation changes, mechanical ventilation, or other administered injury. Inducing mesenchymal sFlt expression at various time points and for varying durations in further experiments may allow us to evaluate the developmental pathophysiology of neonatal lung diseases and the efficacy of potential therapies. This will also enable subsequent studies on the sequelae of these neonatal lung diseases on adult pulmonary structure and function. In this model, VEGF downregulation at birth drives structural and functional abnormalities that persist post-alveolarization and into adulthood. In humans, preliminary data suggest that infants with respiratory complications continue to have respiratory difficulties into adulthood [13]. As a growing number of infants with RDS and other neonatal respiratory diseases survive with improving medical care, investigating the long-term effects of perinatal interventions in viable and pertinent animal models will become increasingly important.

## Supporting Information

S1 File Online Methods Supplement. Additional detail is available regarding pulmonary function tests, tissue morphology, collagen stain, immunofluorescence, and Western blots experiments.
(DOCX)
