# Supplementary material for: Sequestration of Vascular Endothelial Growth Factor (VEGF) Induces Late Restrictive Lung Disease
Source: PLoS One. 2016 Feb 10;11(2):e0148323. doi: 10.1371/journal.pone.0148323 (PMC4749176; doi:10.1371/journal.pone.0148323)
Supplement: S1 File — Additional detail is available regarding pulmonary function tests, tissue morphology, collagen stain, immunofluorescence, and Western blots experiments. (DOCX) [file pone.0148323.s001.docx]

**Sequestration of Vascular Endothelial Growth Factor (VEGF) Induces Restrictive Lung Disease**

Minna M Wieck, Ryan G Spurrier, Dan E Levin, Salvador Garcia Mojica, Michael J Hiatt, Raghava Reddy, Xiaogang Hou, Sonia Navarro, Jooeun Lee, Amber Lundin, Barbara Driscoll, Tracy C Grikscheit

**Online methods supplement**

**Pulmonary function tests**

Mice were anesthetized with a 40mg/kg intraperitoneal (IP) injection of sodium pentobarbital. Following confirmation of adequate anesthesia with toe pinch and 70% ethyl alcohol cleanse, an 18G angiocatheter was inserted into the trachea via anterior cervical incision. Pulmonary function tests were obtained with plethysmography via tracheostomy and forced pulmonary maneuvers (SCIREQ flexiVent, Tempe, AZ). Pressure-volume loop (PV loop), compliance and elastance were compared. Lung volumes were corrected for total bodyweight.

**Tissue Morphology**

Mice were anesthetized with a 40mg/kg IP injection of sodium pentobarbital. Sternectomy was performed to expose the heart and lungs. Slow, right ventricular, intracardiac injection with 20 ml of 1X phosphate buffered saline (PBS) was performed to drain and rinse the pulmonary vasculature. The airway was infused via tracheostomy tubing with 1x PBS at a constant pressure of 25 cmH20 for 2 min. Alveolar expansion was confirmed visually. The trachea was then occluded with a suture tie and the lungs were explanted. Both lungs, en bloc, were fixed in 10% buffered formalin for 24 hours. The left lower lobe was embedded in paraffin.

**Collagen stain**

Collagen was quantified using Fiji ImageJ (32) in the following manner.  Dye colors were separated in the raw RGB images using the color deconvolution function with the following RGB color definition matrix:  color 1 (Sirius red, collagen): 26.863525, 54.626385, 50.577736; color 2 (Sirius green): 23.301447, 16.637552, 16.10618; color 3: 0, 0, 0.  The integrated density of the deconvoluted Sirius red images was measured and converted to optical density (OD) with the formula OD = log10(2048 x 1536 x 255 / integrated density where 2048 x 1536 is the full image pixel area.  Vein lumen cross-sectional areas were measured by selection with the wand tool (tolerance 15.0) in the raw images.

**Immunofluorescence**

Slides were incubated for one hour at room temperature with primary antibodies: Syrian hamster monoclonal antibody to T1α (1:500, Jackson Immunoresearch), surfactant protein C (SPC, 1:500, Seven Hills Bioreagents, Cincinnati, OH), Clara Cell 10 (CC10, 1:250, Santa Cruz Biotechnology), monoclonal anti-actin, or α-smooth muscle-Cy3 antibody (αSMA, 1:300, Sigma). Slides were washed again with TBST and then incubated with fluorescently labeled secondary antibodies: Cy5 goat anti rabbit (1:250, Jackson Immunoresearch), Cy3 goat anti-hamster (1:500, Jackson Immunoresearch) or Cy3 goat anti mouse (1:200, Jackson Immunoresearch). SPC+ AECII counts were performed via computer assisted image processing using ImageJ to quantify the total number of DAPI-positive cells and SPC+ AECII cells per field via segmentation and object-based counting in 20X microscopy fields.

**Western blot analysis**

Mouse monoclonal antibodies to β-actin (ICN Biomedicals, Costa Mesa, CA) and matrix metalloproteinase 9 (MMP9, CHEMICON, Billerica, MA); rabbit polyclonal antibodies to VEGF Receptor 2 (VEGFR2, Cell Signaling Technologies, Beverly, MA), phospho VEGFR2 (pVEGFR2, abcam, Cambridge, MA), alpha smooth muscle Actin (αSMA, abcam), pro-surfactant protein C (pro-SPC, abcam), pro-surfactant protein B (SPB, Seven Hills, Cincinnati, OH), PI3K (Cell Signaling Technologies), AKT (Cell Signaling Technologies), phosphorylated AKT (pAKT, Cell Signaling Technologies), ERK1/2 (Cell Signalling Technologies), phosphorylated ERK1/2 (pERK1/2, abcam), epidermal growth factor receptor (EGFR, Cell Signaling Technologies), phosphorylated EGFR 1 (pEGFR1, abcam), and hypoxia inducible factor 1 alpha (HIF-1α, abcam, Cambridge, MA); and goat polyclonal antibody to CD31 (Santa Cruz Biotechnology Inc., Dallas, TX) were applied at a concentration of 1-2 μg/ml. Horseradish peroxidase-labeled goat anti-mouse IgG and goat anti-rabbit IgG (Sigma) were then applied as secondary antibodies at a dilution of 1:10,000.
